# Role of CNSs Conserved Distal Cis-Regulatory Elements in CD4 + T Cell Development and Differentiation

**DOI:** 10.3389/fimmu.2022.919550

**Published:** 2022-06-20

**Authors:** Xunyi Long, Chen Luo, Zhengming Zhu

**Affiliations:** ^1^ Department of General Surgery, Second Affiliated Hospital of Nanchang University, Nanchang, China; ^2^ Jiangxi Medical College of Nanchang University, Nanchang, China

**Keywords:** human, CNSs, T-cells, gene regulation, cell differentiation

## Abstract

Naïve CD4^+^ T cells differentiate into diverse subsets of effector cells and perform various homeostatic and immune functions. The differentiation and maintenance of these different subsets are controlled through the upregulation and silencing of master genes. Mechanistic studies of the regulation of these master genes identified conserved and distal intronic regulatory elements, which are accessible subsets of conserved non-coding sequences (CNSs), acting as cis-regulatory elements in a lineage-specific manner that controls the function of CD4^+^ T cells. Abnormal CNS activity is associated with incorrect expression of master genes and development of autoimmune diseases or immune suppression. Here, we describe the function of several conserved, distal cis-regulatory elements at the *Foxp3, Rorc, Il-4, Il-10 and Il-17* gene locus were shown to play important roles in CD4^+^ T cells differentiation. Together, this review briefly outlines currently known CNSs, with a focus on their regulations and functions in complexes modulating the differentiation and maintenance of various CD4^+^ T cells subsets, in health and disease contexts, as well as during the conversion of T regulatory cells to T helper 17 cells. This article will provide a comprehensive view of CNSs conserved distal cis-regulatory elements at a few loci that control aspects of CD4^+^ T cells function.

## Introduction

CD4^+^ T cells play an indispensable role in immunity, especially in cellular immunity, by coordinating immune responses during inflammation and autoimmune responses (1). On antigen stimulation, naïve CD4^+^ T cells can differentiate into various subsets of effector T cells, such as T-helper 1 (Th1), T helper 2 (Th2), or T helper 17 (Th17), and regulatory T cells, which involved in cellular immunity or assisting humoral immunity (2). Activated T cells may also differentiate into T regulatory cells specializing in immune suppression and tolerance, which can be divided into three subsets: thymus-derived Treg (tTreg), natural Treg (nTreg) arising from the thymus, and endogenous induced Treg (pTreg) differentiated from antigen-stimulated T cells in peripheral tissues (3), and induce Treg cells (iTreg) differentiated from normal T cells induced *in vitro* by TGF-β (4). The nTreg mainly suppresses the development of autoimmune diseases and raises the activation threshold of the individual immune response, while iTreg cells tend to maintain the non-inflammatory state of tissues, suppresses the immune response against environmental and food allergens, and weaken the inflammatory response. nTreg cells and iTreg cells work together to play a role in maintaining the immune homeostasis (4). Th differentiation and polarization take place in secondary lymphoid organs and is dictated by different signals, including duration and strength of TCR engagement with peptide/MHC class II complexes on antigen presenting cells, co-stimulatory molecules, and cytokines (5, 6). Among the different Th subsets, Th1 cells protect against intracellular pathogens (7). Th2 cells promote humoral immunity and host response to extracellular pathogens, but also allergy and asthma (8). Th17 cells, mainly found in mucosa, protect against extracellular bacteria and fungi, but also participate to autoimmune and inflammatory diseases (9), and T follicular helper cells act in germinal centers, where they promote affinity maturation of the B cell receptors and can contribute to the emergence of lymphomas (10). Essential transcription factors for CD4^+^ T cell differentiation are FOXP3 for Treg (11), RORγt for Th17 (12), TBET for Th1 (13), BCL6 for Tfh (14) and GATA-3 for Th2 cells (15). These lineage-specific master transcription factors determine the fate and function of CD4^+^ T cells and cross-regulate each other’s expression through a dynamic balance. In the past decade, it has become apparent that these master regulators can even be co-expressed, thereby underpinning CD4^+^ T cell heterogeneity and plasticity (6, 16). For example, FOXP3 and RORγt play crucial roles in Treg/Th17 transition. Most recently, the concept of CD4^+^ T cell plasticity has been further investigated by single-cell transcriptomics. This research gave rise to new paradigms representing effector T cell heterogeneity as a transcriptional continuum, progressing from naïve T cells towards increased expression of effector molecules shaping their response to activation and cytokine polarization (17). In the same direction, Kiner et al. proposed that the transcriptional program of CD4^+^ T cell differentiation forms a ‘polarized continuity’ that cannot be resolved into discrete Th cell types (18). Jones and colleagues revealed that activated CD4^+^ T cells redirect their metabolic pathways to generate enough energy to support cellular functions and synthetize the biocomponents necessary to their division and proliferation in response to different challenges like fighting infections, or preventing diseases like cancer, autoimmunity, or allergies (19, 20). These specialized functions are acquired by native CD4^+^ T cells upon activation, through the implementation and stabilization of distinctive transcriptional programs tightly regulated by transcription factors, cis-acting DNA motifs, and chromatin remodeling.

**Figure 1:**
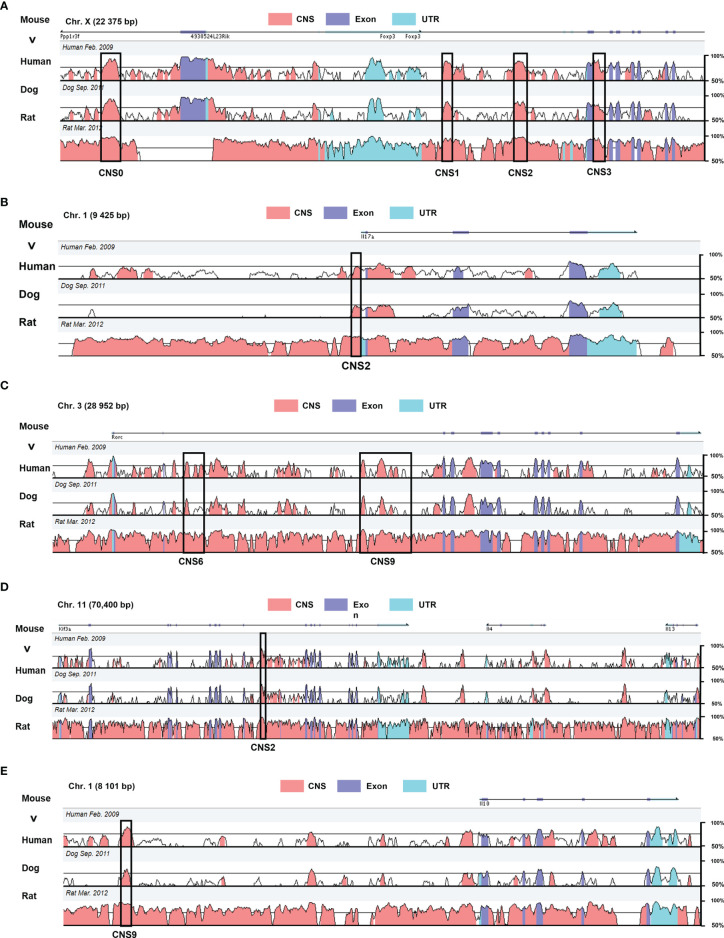
Genomic alignment of the CNSs at major regulatory gene loci in mouse with the homologous sites in human, dog, and rat. The X axis represents the mouse genomic sequences. The Y axis indicates the percentage of identity between different species (minimum cutoff 50%; maximum 100%) **(A)** *Foxp3* CNS0–3 regulating Treg differentiation. **(B)** *Il-17* CNS2 responsible for IL-17 expression. **(C)** *Rorc* CNS6 and CNS9 acting in conjunction with RORγt to promote Th17 differentiation. **(D)** *IL-4* CNS2 regulating Th2 and Tfh differentiation. **(E)** *Il-10* CNS2 impact on Th2 differentiation. These data were obtained from the website VISTA browser and NCBI Gene Database.

Conserved non-coding sequences (CNSs) are DNA regions with high degrees of conservation across species (21, 22). Some accessible subsets of CNSs are conserved and distal cis-regulatory acting motifs recognized by transcription factors and chromatin modifiers activating and maintaining gene expression (23, 24). CNSs are defined as sequences longer than 100 bp with at least 70% of nucleotide identity between mouse, human, dog, and rat genomes, assessed through the VISTA Gateway program (**Figure 1**) (25–27). Some CNSs are cis-regulatory enhancers that are pre-assembled before differentiation and regulate the specific expression and chromatin modification at gene loci (28–30). They also increase the basal transcriptional activity of the gene promoters and transcriptional start sites (TSSs) (31). These enhancers can now be identified by using tiled CRISPR activation (CRISPRa), a method that allows rapid mapping of functional enhancers at target loci (32, 33). In addition, multi-omics and CRISPR/CAS9-mediated editing of human T cells are applied, allowing the identification and validation of distal regulatory elements, starting with genome-wide analysis of the chromatins to identify potential gene enhancers in normal tissues. CRISPR/CAS9 genome editing was then used to validate target regions, revealing the roles of regulatory genes. Finally, potential drug targets for the disease are identified through experimental validation. And this combination of genome-wide association studies (GWAS) and 3D epigenomics facilitates the identification of targets for drug repurposing or compound development (34). Most enhancers boost gene expression from distal locations (35). Upon activation, the naïve CD4^+^ T cells convert the different signals they receive into specific gene expression programs by regulating the abundance, interactions, and positions of master transcription factors (36), thereby acquiring different effector or regulatory lineage identities. CNSs participate in this differentiation by controlling the expression and stability of lineage-specific genes (37). Throughout T cell development, specific CNSs undergo permissive chromatin remodeling, which enable effector T cell differentiation in a lineage-specific manner by governing critical sets of genes. For instance, FOXP3 regulates the growth and activity of Treg cells, and its deletion or malfunction cause severe autoimmune diseases and inflammatory bowel disease (38). Distinctive enhancers contribute to Treg differentiation, which are annotated CNS1, CNS2, and CNS3, relative to their respective distance from *Foxp3* TSS. In addition, *Il-17* enhancers promote IL-17 secretion, which promotes Th17 cell differentiation (39), while *Rorc* is a Th17 cell lineage gene whose distal enhancers, such as CNS6 and CNS9, promote the commitment of Th17 (27). In Th2 cells, CNS9, an enhancer of *Il-10*, increases IL-10 expression (40), and *Il-4* CNS2 induces mainly IL-4 expression in Th2 (41), but *Il-4* CNS2 promotes Tfh differentiation as a specific enhancer in Tfh cells (42). These immune dysfunction-associated noncoding enhancers act specific lineages of genetic programs in CD4^+^ T cell differentiation and are applied to control temporal gene regulation of stimulus response in disease. For example, after IL-2 binding to the IL-2 receptor subunit (IL-2RA), *Foxp3* can be activated to promote Treg differentiation. Deletion or mutation of the IL-2RA enhancer does not completely block IL-2RA expression but delays the time of activation of *Foxp3*, shifting the polarization of naïve T cells towards pro-inflammatory TH17 and away from Treg. This delayed regulatory approach regulates the binding of CNSs on specific lineage genes to transcription factors that influence the direction of CD4^+^ T cell differentiation and control disease (32).

**Figure 2:**
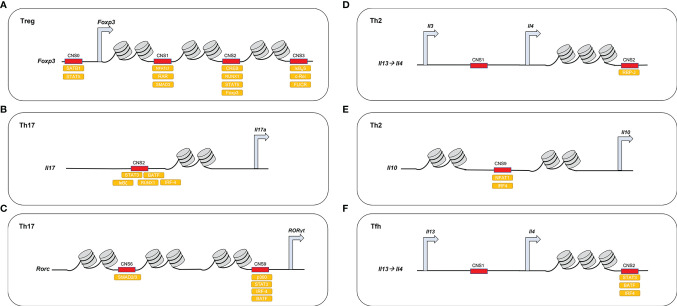
DNA-binding proteins access demethylated CNSs in different CD4^+^ T cell lineages. **(A)** In Treg, *Foxp3* CNS1–3 are demethylated and recruit different activating molecules. *Foxp3* CNS0 recruits SATB1, STAT5; *Foxp3* CNS1 recruits NFATc1, SMAD and RAR; *Foxp3* CNS2 recruits CREB, STAT5, RUNX1, FOXP3; and *Foxp3* CNS3 recruits c-REL, IκB**_N_** S, FLICR. **(B)** In Th17, *Il-17* CNS2 recruits STAT3, IRF-4, RUNX1, BATF1, and IκBζ. **(C)** In Th17, *Rorc* CNS6 acts downstream of the TGF-β-SMAD pathway and CNS9 recruits STAT3, p300, IRF4, and BATF. **(D)** In Th2, *Il-4* CNS2 acts downstream of the Notch/RBP-J pathway. **(E)** In Th2, *Il-10* CNS9 recruits NFAT1 and IRF4 **(F)** In Tfh, *Il-4* CNS2 recruits BATF1, IRF4, and STAT3.

The activity of some of the CNSs regulating master transcription factors and lineage-specific cytokines have become indicators of CD4^+^ T cell lineage identity and are reviewed in this manuscript (**Figure 2**). In addition, this review discusses the dysfunctions of these CNSs in inflammatory or autoimmune contexts, their regulation, as well as possible intervention for the management of these diseases. While this article concentrates mainly on the CNSs that regulate *Rorc* and *Foxp3*, in particular during the conversion between pro-inflammatory Th17 and anti-inflammatory Treg phenotypes (43, 44), we also provide a more general overview of the latest findings on the functions and underlying mechanisms of CNS regulation in different effector T cell subsets (**Table 1**).

**Table 1:** CNS functions in CD4^+^ T cells.

Gene	CNSs	Cell type	Binding molecules	Functions	Ref.
*Foxp3*	CNS0	Treg	SATB1, STAT5	Super-enhancer activating multiple CNSs demethylation	(45)
	CNS1	Treg	NFATc1, SMAD and RAR	Induces peripheral Treg, especially in intestinal lymphoid tissue	(26)
	CNS2	Treg	CREB, STAT5, RUNX1, FOXP3	Stabilizes *Foxp3* expression in Treg	(26)
	CNS3	Treg	c-REL, IκB**_N_** S, FLICR	Facilitates epigenetic modification and induces *Foxp3* promoter remodeling in naïve cells	(26)
*Il-17*	CNS2	Th17	STAT3, IRF-4, RUNX1, BATF, IκB**ζ**	Promotes Th17 differentiation and IL-17 expression	(39)
*Rorc*	CNS6	Th17	TGF-β-SMAD pathway	Th17 commitment	(27)
	CNS9	Th17	STAT3, p300, IRF4, BATF	Promotes Th17 differentiation	(27)
*Il-4*	CNS2	Th2	NOTCH-RBP-J pathway	Induces IL-4 expression	(41)
*Il-10*	CNS9	Th2	NFAT1, IRF4	Increases IL-10 expression	(40)
*Il-4*	CNS2	Tfh	BATF, IRF4, STAT	Tfh-specific enhancer	(42)

CNSs, conserved non-coding sequences; Th, T helper cells; Treg, T regulatory cells.

## CNSs Involved in CD4^+^ T Cells Differentiation

### CNSs Is Important for Treg Cells Development

#### *Foxp3* CNS0 in Treg Cells

The CNS0 of the *Foxp3* gene is located 8 kb upstream of its TSS and is hypomethylated in Tregs (**Figure 1A**) (46). The binding of SATB1, a global genome organizer (47), to different *Foxp3* CNSs, including CNS0, dictates Treg fate (**Figure 2A**; **Table 1**) by increasing chromatin accessibility at the *Foxp3* locus. Yohko et al. (45) identified CNS0 as a Treg super-enhancer (SE) with high density of acetylation at lysine 27 of the histone 3 proteins (H3K27ac) in mouse Treg cells, which binds an abundance of TF and chromatin remodelers regulating this lineage (48–51). CNS0 is activated first in T precursor cells, where it initiates a Treg-specific transcription program. Satb1 deficiency impairs CNS0 activation in human Treg cells, due to decreased demethylation, and consequently, *Foxp3* activation, which might originate autoimmune diseases (47, 52). Marc et al. proposed a more complex mechanism of *Foxp3* activation than the sole binding of SATB1 to CNS0 (53). Their model involves intermediary factors, such as Ten-eleven translocation enzyme (TET) or DNA methyltransferase (DNMT), apparently necessary to achieve Treg commitment. A recent study discovered that IL-2 triggers the binding of STAT5 to CNS0, thereby increasing *Foxp3* expression in mouse genetic model. The same study showed that CNS0 deficiency causes impaired Treg cell production in neonates, which becomes partially compensated with age through an increase in cell-intrinsic mechanism of activation. However, CNS0 deficiency combined with impairment of other gene functions, such as *Aire* deficiency, causes severe Treg cell deficit, and markedly exacerbated autoimmune disorders. Thus, CNS0 ensures Treg differentiation and minimizes the risks of autoimmunity (54). Further, CNS0 and CNS3, another regulatory sequence in *Foxp3*, work in hierarchical and independent ways: CNS0 relies on SATB1 and the IL-2/STAT5 signaling, while CNS3 depends on c-REL in mouse (55). FOXP3 has been found to be essential for maintaining immune self-tolerance by regulating the development and function of thymus Treg cells (56). CNS0 serves as a key unit of enhancer cluster by bringing together individual enhancers and the *Foxp3* promoter in three-dimensional chromatin space and providing a platform for multiple TF complexes to ensure stable control of Foxp3 expression (55). Thus the manipulation of CNS0 could modulate the production and quality of Treg in clinical treatment. Future intervention strategies targeting CNS0 may help boost Treg production *in vitro.*

#### *Foxp3* CNS1 in iTreg Cells

Like CNS0, CNS1 works as an enhancer at the *Foxp3* locus (**Figure 1A**) and mediates Treg differentiation and functions (26). This enhancer was first identified by Tone et al. who demonstrated its acetylation by NFATc1 and SMAD3 and its role in maintaining stable *Foxp3* expression in mouse (57). CNS1 is crucial for iTreg differentiation, particularly in peripheral tissues and gut, but dispensable for nTreg differentiation. Further, CNS1-independent nTreg co-express RORγt, which is upregulated under inflammatory conditions in mouse. Thus, RORγt expression and CNS1 dependency are distinctive marks of iTregs and nTregs identities (58). CNS1 regulates *Foxp3* expression by recruiting NFATc1, SMAD3, and retinoic acid receptor (RAR) (**Figure 2A**) (57, 59, 60). CNS1 is activated upon TGF-β exposure (61), which induces SMAD2/SMAD3 binding, *Foxp3* transcription, and nTreg differentiation (62, 63).

Treg differentiation is facilitated by the binding of CNS1 by RAR, a metabolic regulator balancing Treg versus Th17 differentiation that increases CNS1 acetylation in mouse (64). In addition, the SET and MYND Domain 3 (SMYD3) protein, a SET histone methyltransferase, regulates the TGF-β/SMAD3 pathway by primary epigenetic activation of CNS and is also involved in Th17 regulation in mouse (65). Further, serum and glucocorticoid-inducible kinase 1 (SGK1) modulates Treg versus Th17 development by modulating FOXO1 phosphorylation and its nuclear exit. Failure of FOXO1 to bind CNS1 inhibits Foxp3 expression in mouse, causing pro-inflammatory Th17 differentiation during autoimmune inflammation (66).

CNS1 is mostly active in iTregs, which contribute to the resolution of immune responses against pathogens infecting the lungs and stomach of mouse, and prevent immune pathologies caused by inflammation (67). For example, respiratory syncytial virus infection is more severe in SMYD3-deficient than in wild-type mouse, due to lower numbers of Tregs. SMYD3 regulates Foxp3 expression through a TGFβ1/Smad3-dependent mechanism, and its deletion leads to a reduction in H3K4me3 of foxp3-patterned CNS1, which affects iTreg cell formation. And the resulting lack of control on pro-inflammatory cytokines boosts IL-17 production, allowing SMYD3-/- mice to exhibit exacerbation of RSV-induced disease (65, 68). The resulting lack of control on pro-inflammatory cytokines boosts IL-17 production. Based on *Foxp3* CNS1 similarities in human and mouse (69), it is plausible that *FOXP3* CNS1 plays a role in the control of human infectious diseases.

Recent studies have suggested that TGF-β may be important for differentiation of both thymus and peripheral Treg cells by inducing the activation of the Foxp3 promoter in EL-4 cells (57). Furthermore, mouse *Foxp3*-4kb sequence devoid of CNS1 was lacking enhancer activity whereas incorporation of CNS1 into zebrafish or opossum Foxp3-4kb sequence reconstituted enhancer activity (70). Moreover, an intronic Foxp3 enhancer CNS1, that contains Smad3 and RAR (retinoic acid receptor) binding sites, facilitates TGF-β-dependent Foxp3 induction and pTreg cell differentiation (26). That means CNS1 is responsible for TGF-β-dependent induction and maintenance of Foxp3 expression. and intervenes in TGF-β-mediated regulation of maternal-fetus tolerance in mouse (70). That is, CNS1 deficiency causes mucosal Th2-mediated inflammation and abortion in mouse, implying that CNS1 maintains Treg identity to improve tolerance (46, 71). Furthermore, iTregs suppress immune response in the gut during microbiota colonization and regulate the metabolic function of the gut microbes in mouse (72). A comparison between CNS1-/- and CNS+/+ iTregs demonstrated a lack of CNS1-/- iTreg affects gut homeostasis in mouse by disturbing the establishment of the microbiota or changing the microbial communities over time (73). These results imply that iTregs participate in shaping the composition and function of the microbial community (72). Drugs that affect gut bacteria may boost Treg differentiation. Arpaia et al. (74) uncovered that butyrate and propionate, both produced by commensal bacteria, boost the differentiation and number of extrathymic CNS1-dependent iTreg in mouse, implying that the microbial metabolism links the microbiota and the immune system to mitigate inflammation. Clarissa (75) used CNS1-deficient mouse to demonstrate that the bile acid 3-b-hydrodeoxycholic acid (isoDCA) increases Treg number in the colon by inducing CNS1-dependent *Foxp3* expression. Hence, the microorganisms producing isoDCA promote iTreg generation, further linking the microbial metabolism to beneficial effects on immune homeostasis. However, *Foxp3* CNS1 mutation could be compensated by increased induction of anergic (Tan) cells of mouse and subsequent conversion of Tan cells into iTreg cells promoted by commensal bacteria, thereby preventing gut inflammation and autoimmunity (76).

Collectively, these studies have been centered on the control and maintenance of Treg identity by *Foxp3* CNS1. CNS1 is required for iTreg differentiation, particularly in the gut, downstream of the TGF-β/SMAD3 pathway. Consequently, CNS1 represents a target to modulate gut iTreg and constitutes a molecular link between microbial metabolites and immune regulation. Thus, CNS1 could serve as therapeutic target to intervene on gut iTreg and balance pro- and anti-inflammatory signals for the management of gut immune responses. More research on the mechanisms of iTreg control by *Foxp3* CNS1 is essential to explore new directions for treatment of immunological diseases in the gut. For instance, manipulating the microbiota or its metabolism may influence the differentiation and stability of iTregs through CNS1 regulation.

#### *Foxp3* CNS2 in nTreg Cells

*Foxp3* CNS2 (**Figure 1A**), also called the Treg-specific demethylated region, was first characterized by Zheng et al. (26). CNS2 is an enhancer that maintains stable expression of *Foxp3* in mature Tregs during cytokinesis and pro-inflammatory conditions (77, 78). Recently, Cameron et al. used CRISPR/dCas9 to induce constitutive *Foxp3* transcriptional activation in iTreg, which caused CNS2 demethylation and improved the stability and suppressive functions of the Tregs in mouse colitis (79). TCR stimulation with sufficient intensity is a critical determinant to induce *Foxp3* CNS2 demethylation (80). CNS2 with abundant CpG islands becomes fully demethylated in mature Tregs to maintain *Foxp3* expression (81). CNS2 is activated through the binding of multiple transcription factors, including CREB, STAT5, RUNX1/CBF, and FOXP3 (**Figure 2A**) (82). The interaction of CNS2 with CREB and STAT5 is essential for *Foxp3* expression (83). CREB binds and demethylates CNS2 to increase *Foxp3* expression, while CpG island methylation reduces CREB ability to bind the *Foxp3* locus in mouse (84). Upon IL-2 exposure, STAT5 binding to CNS2 drives proliferation and various Treg functions (85, 86). Therefore, during the division of mature cells, CNS2 acts downstream of IL-2/STAT5 signaling to enable heritable *Foxp3* activation. In inflammatory cytokine milieu lacking IL-2, CNS2 recruits STAT5 to sustain *Foxp3* expression and Treg activity in mouse, thereby suppressing organ-specific chronic autoimmune inflammation (87, 88). In contrast to STAT5, the IL-6/STAT3 axis downregulates *Foxp3* and up-regulates *Rorc*, skewing T cell differentiation towards TH17 fate in mouse (89). RUNX1/CBFβ heterodimers bound to CNS2 (90, 91) up-regulate *Foxp3* and uphold Treg-mediated immune homeostasis (57). Moreover, the lack of inducible-co-stimulator (ICOS), a context-dependent regulator, reduces CNS2 demethylation and *Foxp3* expression in mouse, demonstrating the requirement of ICOS for Treg lineage stability (92). Upon CNS2 demethylation, FOXP3 binds CNS2 and maintains a positive regulation loop (26). Further, TET enzymes catalyze the hydroxylation of DNA 5-methylcytosine (5-mc) at the CNS2 locus, engaging its demethylation in mouse (52). Two members of the TET family, TET2 and TET3, recruit transcription factors like NFAT at the CNS2 CpGs to increase Foxp3 demethylations (77, 93, 94). Consistent with these studies, Nakatsukasa et al. discovered that TET shortage increases IL-17 and reduces Foxp3 expression in mouse. In iTreg, TET is more crucial to CNS2 demethylation than in nTreg (95). The fact that Tet-deficient mouse developed autoimmune disorders further demonstrates the significance of TET for Treg identity (96, 97). Single nucleotide polymorphisms associated with autoimmune diseases that affect Treg functions are abundant in the CNS2 region (98, 99).

Recent studies focused on elucidating how CNS2 status in FOXP3^+^ Treg influenced the development and function of Tregs. Vitamin C increases TET activity and the TGF-β level in mouse, which favors *Foxp3* expression. Yue et al. suggested that Vitamin C could promote iTreg development in mouse by promoting CNS2 demethylation by the TET enzymes in clinical setups (100, 101), especially in hypoxic conditions (102). Takahashi et al. demonstrated that in mouse CNS2 can recruit SOCS1, a negative cytokine regulator, causing its demethylation and allowing for sustained *Foxp3* expression to preserve the stability and integrity of Treg identity (103). BLIMP-1 counteract IL-6/STAT3-mediated suppression of Treg identity by preventing CNS2 methylation by the DNA methyltransferase 3 alpha (DNMT3a), and therefore could represent a drug target in inflamed non-lymphoid tissues in mouse (104). *Foxp3* CNS2 methylation in iTreg stabilizes the stability of this T cell subset (105–107). However, some CNS2 CpG islands remain methylated in iTreg, indicating unstable expression of *Foxp3* in these cells (108–110). In addition to DNA methylation, histone epigenetic modifications also modulate CNS2 activity. Trans-retinoic acids, which up-regulate ERK, exert a synergistic effect with TGF-β and up-regulate histone H3K4 methylation at the CNS2 locus in mouse, thereby maintaining *Foxp3* transcription. Trans-retinoic acids could help prime iTreg before treatment of autoimmune diseases or organ transplantation (111). Further, liver kinase b1 (LKB1), a tumor suppressor, prevents STAT4-mediated CNS2 methylation partly through NF-κB inhibition in mouse, and therefore, stabilizes *Foxp3* expression (112). Thus, LKB1 up-regulation in Tregs could constitute a therapeutic target for autoimmune diseases. Another study from Chen et al. using a model of neonatal Treg-mediated protection in cardiac allograft transplantation showed that CNS2 demethylation prolonged the time of heart survival in mouse (113). Therefore, neonatal Treg cultured *in vitro* could provide a new method to modulate immune responses. To achieve a stable Treg lineage with a high level of *Foxp3* expression, Chen et al. used three types of chemical chromatin-modifying complexes, which bind and fully demethylate CNS2. In models of transplant and graft rejection in mouse, adoptive transfer of these manipulated iTregs could prevent graft rejection and experimental autoimmune encephalomyelitis (EAE), thereby prolonging survival time. These studies demonstrate that *Foxp3* CNS2 stabilizes therapeutic Tregs generated *in vitro* (114). In active ankylosing spondylitis, *Foxp3* CNS2 is hypermethylated, causing Treg dysfunction, which opens a therapeutic opportunity by targeting CNS2 in this disease in human (115). Recently, Li et al. (116) investigated the possibility to induce a Treg phenotype *in vitro* by histone acetylation at *Foxp3* CNS0 and CNS3 loci to induce Foxp3 transcription, and subsequent demethylation of the CNS2 to stabilize Foxp3 transcription, with TET playing a major role in mouse. These processes reveal a stepwise mechanism of iTreg specification. Moreover, Okada et al. (117) edited the epigenome of mouse at the *Foxp3* locus to demethylate CNS2, which slightly stabilized Foxp3 expression in an inflammatory context. Kressler et al. used CRISPR-Cas9 to demethylate CNS2 selectively by using human and mouse primary T cells (118). They hypothesized that CNS2 demethylation was necessary for Foxp3 induction but found that other key events were necessary for Treg induction *in vitro.* Thus, this intervention alone could not induce a stable and fully functional Treg phenotype. Overall, the CNS2-dependent stability of the Treg lineage is essential to prevent organ-specific autoimmunity, non-spontaneous chronic inflammatory conditions, and metabolic inflammation. Thus, the stabilization of Treg identity through *Foxp3* CNS2 manipulation could be exploited to generate new therapeutic applications and improve current treatments.

#### *F*oxp3 CNS3 in iTreg cells

Zheng et al. firstly defined *Foxp3* CNS3 as an intronic regulatory element facilitating epigenetic modifications, such as Histone H3 lysine K4 methylation (H3K4me) and Histone H3 lysine K27 acetylation (H3K27ac) (**Figure 1A**) (26). During the development and differentiation of iTreg, CNS3 takes over CNS1 and CNS2 functions and promotes *Foxp3* expression. CNS3 induces *Foxp3* promoter remodeling and opening in Treg progenitors, rendering them responsive to a wide range of TCR stimulations, even with low intensity in mouse (119). Mutations within mouse *Foxp3* CNS3 severely impairs Treg development, while immune tolerance relies on a CNS3-dependent mechanism that controls CD4^+^ T cell responses (120). Upon TCR and CD28 stimulation, CNS3 recruits pioneer c-REL homodimers, which leads to demethylation and *Foxp3* transcription (46, 56, 121). In addition, IκB_NS_, an atypical IκB protein, and p50 can bind CNS3 and modulate *Foxp3* expression. During chronic inflammation, IκB is a key *Foxp3* inducer, thereby contributing inflammation dampening in mouse (122). Long et al. showed that NF-κB could also bind *Foxp3* CNS3 and mediate its demethylation in mouse, resulting in *Foxp3* expression (123). In addition, TRAF6, an NF-κB activator, maintains *Foxp3* expression (124).The binding of c-REL and IκB is regulated by NF-κB (**Figure 2A**) (122). Furthermore, *Foxp3* CNS3 is affected by metabolites of bile acid, which impacts on Treg differentiation. For instance, the lithocholic acid (LCA) derivative IsoalloLCA can promote Treg differentiation by raising the H3K27ac level at the *Foxp3* CNS3 locus in mouse, while another derivative, 3-oxoLCA, binds to RORγt to reduce Th17 differentiation in mouse lamina propria. These molecules represent novel regulatory pathways for balancing Th17 versus Treg differentiation in autoimmune diseases and inflammation (125). However, the long noncoding RNA (lncRNA) *Flicr* (*Foxp3* long intergenic noncoding RNA), specifically expressed in mature Treg, operates as a negative regulator of chromatin accessibility in the CNS3 region and limits Treg activity, contributing to autoimmune diabetes in mouse (**Figure 2A**) (78). Forstnerič et al. targeted CNS1–3 in mammal Hek293 and Jurat cells from mouse using CRISPR technology and assessed the significance and potency of these enhancers for *Foxp3* transcription. CNS3 showed the most potent effect, indicating that it constitutes a useful target to regulate Treg induction (126). CNS3 could also be inhibited to reduce Treg cell formation in cancer and boost antiviral defenses during infections.

In summary, several interventions targeting *Foxp3* CNS could help manipulate Treg in medical setups. First, metabolites or nutriments can act on CNSs to increase iTreg expression. For example, Bacterial metabolites, like isoDCA, could suppress intestinal inflammation by inducing iTreg by interacting with CNS1 (75), and LCA derivatives could promote Treg differentiation by enhancing CNS3 demethylation during inflammation. Vitamin C, which increases TET enzyme activity and promotes CNS2 demethylation, could be used to maintain *Foxp3* expression and Treg development. Second, CRISPR technology could help act on CNSs *ex vivo* in immunotherapy setups. For example, Kressler et al. achieved selective CNS2 demethylation in the endogenous chromatin environment of living cells using a CRISPR-Cas9-TET1-mediated transient transfection epigenetic editing approach. The induced CNS2 demethylation remained stable during T cell clonal expansion in culture, even after the expression of the editing complex had stopped. Although currently this technology does not ensure Treg suppressive activity *in vivo*, future development may allow the reprogramming of patients’ T cells *ex vivo* and reinfusion of effector T cells converted into stable regulatory T cells (118).

Overall, *Foxp3* CNSs could be targeted to develop complementary therapies and promote Treg cell functions in transplantation, autoimmune diseases, and chronic inflammation.

### CNSs Is Important for Th17 Cells Development

#### *Il-17* CNS2 in Th17 Cells

CNS2 is a main regulator of the *Il-17* gene, the expression of which characterizes Th17 cells. CNS2 contains a binding site for proteins of the ROR family and was identified 5 kb upstream of the *Il-17* promoter in *Mus musculus* (**Figure 1B**) (127–129). RORγt and its related factor RORα are key Th17 lineage-specific transcription factors and are responsible for the induction of IL-17A and IL-17F expression. Initially, high levels of RORγt and RORα are induced by TGF-β and IL-6 signaling pathways (39, 130, 131). While RORγt combined with RUNX1 at the *Il-17a* CNS2 locus promotes IL-17 expression, the binding of RUNX1 combined with FOXP3 at this site inhibits IL-17 and favors Treg differentiation. However, this competition is alleviated by IL-6 and TGF-β, which inhibit Foxp3 and stabilize RUNX1 and RORγt occupation at the *Il-17a* CNS2 locus in mouse (128, 132). Thus, *Il-17* CNS2 controls the balance between pro-inflammatory Th17 cells and anti-inflammatory Treg cells by regulating the interplay between FOXP3, RUNX1, and RORγt. Th17 cells are responsible for the development of experimental autoimmune encephalomyelitis (EAE) in mouse. Deficiency in basic leucine zipper transcription factor ATF-like (BATF), an inhibitor of Activator protein 1 (AP1) that binds *Il-17* CNS2, prevents Th17 cell differentiation, suggesting that BATF could be targeted to inhibit EAE (133). Moreover, IκBζ, a family member of nuclear IκB, coupled with the ROR nuclear receptors, acts directly on CNS2 to boost its activity and promote Th17 differentiation. Defect in IκBζ confers resistance to EAE in mouse (134). Hence, CNS2 could regulate *Il-17a* and *Il-17f* transcription driven by RORγt in Th17 cells, through the modulation of TLR, STAT3, IRF-4, RUNX1, BATF, and IκBζ signaling (**Figure 2B**) (132–135). CNS2 is regarded as a necessary binding site for RORγt to stimulate IL-17 expression in Th17 cells. However, there is a debate regarding which of the CNSs located at the *Il-17* locus is prominent for the coordination of STAT3, RORγt and Runx1 activities responsible for *Il-17* transcriptional enhancement. Most studies indicate that CNS2 is key; however, Thomas et al. suggested that another CNS, located ~28 kb downstream of the *Il-17* gene, could recruit STAT3, RORγt, and RUNX1 to enhance *Il-17* transcription, and primarily coordinates RORγt activity in mouse (136). Thus, the roles of CNSs at the *Il-17* locus require further study. IL-17 produced by Th17 (137) is crucial for host cells to fight against external pathogens but also promotes autoimmune inflammation, making Th17 a double-edged sword in immunity. Additional research should be conducted to manipulate Th17 production in pathogenic setups. Small molecules may act on the binding factors of CNS2 to suppress pro-inflammatory IL-17 expression. Other CNSs located at the *Ill7-Il17f* locus need further investigation to understand the control IL-17 expression.

The conversion of anti-inflammatory Treg into pro-inflammatory Th17 cells mainly involves transcription factors regulating the expression of the Treg-specific protein FOXP3 and the Th17-specific protein RORγt. RAR and SMYD3 can bind *Foxp3* CNS1 to inhibit *Foxp3* expression and thus enable conversion into Th17 cells. In contrast, bile acid metabolites can regulate this conversion bi-directionally by affecting *Foxp3* CNS3 and *Rorc* locus. In addition, *Il-17* CNS2 can regulate the interaction between FOXP3, RUNX1 and RORγt to control the balance between Treg and TH17 cells: RORγt binding RUNX1 at *Il-17a* CNS2 promotes IL-17 expression, while FOXP3 binding inhibits *Il-17* transcription. Thus, it should be possible to control inflammatory responses by promoting Th17 conversion into anti-inflammatory Treg cells.

#### *Rorc* CNS6 in Th17 Cells

Chang et al. (27) first identified CNS6 at the *Rorc* locus and discovered that its loss increases IL-10 and FOXP3 expression while decreasing RORγt expression in mouse. Studies on *Rorc* CNS6 have focused primarily on its role in Th17 cells and have demonstrated that *Rorc* CNS6 deficiency confers resistance to EAE in mouse (27). RORγt (**Figure 1C**), one of the transcription factors encoded by the *Rorc* gene and upregulated by IL-6 and TGF-β, regulates Th17 development (39, 138). CNS6 activity is also controlled by the IL-6/STAT3 pathway in mouse (139), through direct binding of STAT3 to CNS6 and subsequent chromatin remodeling at the *Rorc* locus (**Figure 2C**). *Rorc* CNS6 is also the principal cis-acting element downstream of the TGF-β signaling pathway, through the recruitment of SMAD or c-MAF in mouse (140). This pathway mediates the regulation of RORγt expression by TGF-β (141). In addition, RAR can bind CNS6 to modulate the effect of TGF-β in mouse (64). Although *Rorc* CNS6 is a main target of the TGF-β/SMAD signaling pathway for the control of RORγt production in Th17 cells, thus far, its partial analysis only demonstrated a role in chromatin regulation but not in *Rorc* promoter activation. Therefore, further research on the roles of CNS6 is required. Th17 cell development could be influenced by targeting the TGF-β/SMAD/CNS6 axis in autoimmune diseases, for CNS6 directly act on TGF-β-induced RORγt expression, which impacts the differentiation of Th17. Thus, methylating or modifying histones at *Rorc* CNS6 may contribute to alleviating autoimmune disease and anti-inflammation.

#### *Rorc* CNS9 in Th17 Cells

Chang et al. (27) first identified *Rorc* CNS9 (**Figure 1C**) as a predominant cis-acting element for Th17 differentiation, as compared to *Rorc* CNS6. Epigenetic activation of the *Rorc* requires CNS9 that recruits STAT3 with strong affinity and is a main target element downstream of the IL-6/STAT3 cascade (**Figure 2C**). Moreover, *Rorc* CNS9 in Th17 has been proven to interact with IRF4 and BATF, both of which are induced by TCR signaling and are highly expressed in Th17 cells, where they modulate RORγt expression in mouse (5, 27). Visel et al. discovered that the binding of p300, a histone acetyltransferase, predicts CNS enhancer activity in mouse (29). Consistent with the role of CNS9 as an enhancer of *Rorc* expression, Chang et al. demonstrated strong interactions between *Rorc* CNS9 and p300 (27). Moreover, the development of pathogenic Th17 cells *in vivo* depends on CNS9, and less on CNS6. *Rorc* CNS9-deficient mouse have high resistance to EAE induction (27). Overall, *Rorc* CNS9 is a main regulatory region that controls the chromatin accessibility of the entire *Rorc* locus. Its methylation could help control Th17 differentiation in autoimmune diseases.

### CNSs Is Important for Th2 Cells Development

#### *Il-4* CNS2 in Th2 Cells

Distinct but overlapping mechanisms operate in Th2 cells to promote IL-4 production (142, 143). A CNS2, also called HS V, has been identified as a primary regulator of the *Il-4* locus, and is located ~10 kb downstream of the *Il-4* locus in mouse (144) (**Figure 1D**). IL-4 acts through STAT6 and drives naïve CD4^+^ T cell differentiation towards Th2, controlled by the transcription factor GATA3 (36). An *Il-4* CNS2 has been identified that is crucial for IL-4 expression, a cytokine responsible for IgG and IgE antibodies production by B cells, the defense against extracellular parasites, particularly helminthic infections, and allergic reactions. Notch/RBP-J interact with *IL-4* CNS2 to drive IL-4 expression and Th2 commitment. A secondary pathway, involving the binding of STAT6 to Il-4 CNS2, also promotes *Il-4* expression and Th2 differentiation in mouse (**Figure 2D**) (41). Recently, Kubo proposed that *Il-4* CNS2 could have distinct activities in Th2 cells (142).

#### *Il-10* CNS9 in Th2 Cells

IL-10 is a cytokine produced by Th cells, Treg, and B cells that are able to suppress immune responses during inflammation and autoimmune diseases, and especially inflammatory bowel disease in mouse (40, 145). By studying Th2 cells, Lee et al. identified a CNS9 element 9 kb upstream of the *Il-10* gene (**Figure 1E**). Their study revealed that *Il-10* CNS9 enhancer activity in Th2 was mediated by NFAT1 and IRF4 (**Figure 2E**), which are two transcription factors upregulated upon TCR stimulation and boost IL-10 expression. Moreover, So et al. (146) demonstrated that 6-Methoxyflavone inhibits *Il-10* CNS9 activity by preventing NFAT1 nuclear translocation and disrupting its binding to this cis-regulatory element in mouse, ultimately causing IL-10 down-regulation. This mechanism reduces IL-10 production in activated T cells (146). IL-10 is a potential therapeutic target in various immune diseases due to its anti-inflammatory functions. Targeting *Il-10* CNSs with drugs or manipulating bacteria could increase IL-10 production for therapeutic purposes. Additional research will clarify the role of other *IL-10* CNSs in Th2 cells.

### CNSs Is Important for Tfh Cells Development

#### *Il-4* CNS2 in Tfh Cells

In Tfh cells, CNS2 is located ~10 kb downstream of the *Il-4* locus in mouse (42) (**Figure 1D**). IL-4 secreting Tfh cells differ from Th2 in that they respond to cognate peptide/MHC complexes and ICOS ligands on B cells in mouse, and participate to the germinal center reaction (147). Harada et al. (42) revealed that *Il-4* CNS2 is a specific enhancer for Tfh cell differentiation, potentially acting downstream of the SLAM/SAP pathway (**Figure 2F**). In addition, BATF, together with IRF4 and STAT, binds to *Il-4* CNS2 to trigger IL-4 expression and boost Tfh cell differentiation, which prevents allergic asthma and peripheral lymphomas (147, 148).

In conclusion, IL-4-producing Tfh can have both beneficial functions and pathogenic effects. On the one hand, IL-4 derived from Tfh helps protective B cell responses in lymphoid organs, partly by promoting IgG1 and IgE production and effective germinal center reactions resulting in high-affinity antibodies in mouse (149). *Il-4* CNS2 is demethylated and activates IL-4 expression in Tfh independently of GATA-3. On the other hand, BATF/IRF4 complexes bound to *IL-4* CNS2 have been implicated in IL-4 transcription in Tfh cells involved in allergic reactions in mouse. Therefore, BATF could be targeted in Tfh cells. As a proof of concept, mutations within BATF prevents the development of allergic asthma for Batf promotes the production of pro-allergic IL-4 by Tfh cells in mouse (150).

## Conclusions and Prospects

In conclusion, CNSs in effector CD4^+^ T cells, especially in Treg and Th cells, play different roles depending on their location at key regulatory gene loci. The regulation of CNSs differs in effector CD4^+^ T cells, especially in Treg and Th cells. CNSs in effector T cells are implicated in a variety of processes, involving differentiation, cytokine production, and gene expression. The main function of the CNSs are to recruit specific transcriptional regulators and chromatin modifiers that control the expression of genes important for cell differentiation and status. Effector T cells modulate immune suppression and release anti-inflammatory cytokines to alleviate diseases, including autoimmune diseases, allograft rejection, allergies, and cancer, where they could be targeted to develop new therapies. There are already many examples of CNSs can affect CD4^+^ T cells identity and differentiation, and conserved, distal cis-regulatory elements at gene locus were shown to play important roles in Treg differentiation. Understanding the exact functions and mechanisms whereby cis-acting elements regulate gene activity need further elucidation of CD4^+^ T cells plasticity could potentially lead to identification of new therapeutic targets for immune-mediated diseases. In particular, CNSs need to be more systematically mapped and identified. Other putative CNSs involved in *Rorc* gene regulation also need further investigation in Th17 cells. Using transcriptomic and epigenetic tools, Yoshida et al. mapped the cis-regulatory elements intervening in the mouse immune system. This study provided information on the global transcriptional regulation of immune cell differentiation and suggested that most TFs positively affect chromosome accessibility, prompting the hypothesis that chromatin opening is the primary mode of control of gene expression of the immune system. For example, most TFs bind *Foxp3* CNSs to control Treg differentiation (151). Potential interventions for the control of autoimmunity at CNS level are multiple and should be studied in greater depth. Oral treatments with drugs or food supplements could be useful. For example, vitamin C could enhance TET enzyme activity to increase Treg production (101). Other strategies could involve manipulating microbiota (76) and their metabolism to trigger CNS activity in gut CD4^+^ T cells and alleviate intestinal inflammation. Further, CRISPR-mediated knock-in, deletions, or chromatin editing at CNSs could help up-regulate important regulatory genes in CD4^+^ T cells and improve their effectiveness in adoptive transfer-based T cell therapies (118). However, currently, technical limitations around CNS targeting still exist. One limitation is that small molecules acting on CNSs drive both anti- and pro-inflammatory cell differentiation, leading to uncontrolled immune reactions. Therefore, safety evaluations should be performed before translating these discoveries to clinics. Finally, for CNSs to provide novel targets and strategies, it is necessary to explore the mechanisms whereby CNSs could be manipulated to produce sufficient and stable effector CD4^+^ T cells relevant to the treatment of immune diseases and cancer.

## Author Contributions

CL and ZZ designed the study, provided suggestions for the project. XL wrote the manuscript, analyzed the data, CL revised the figures and tables. ZZ provide funding acquisition. All authors contributed to the article and approved the submitted version.

## Funding

This work was supported by a project from Jiangxi Provincial Education Department of Science and Technology Research (Nos. 180075).

## Conflict of Interest

The authors declare that the research was conducted in the absence of any commercial or financial relationships that could be construed as a potential conflict of interest.

## Publisher’s Note

All claims expressed in this article are solely those of the authors and do not necessarily represent those of their affiliated organizations, or those of the publisher, the editors and the reviewers. Any product that may be evaluated in this article, or claim that may be made by its manufacturer, is not guaranteed or endorsed by the publisher.
